# Effectiveness Outcomes from a Social Network Diffusion Intervention to Increase COVID-19 Testing and Vaccination among Individuals Impacted by the Criminal Legal System in Urban U.S. Cities

**DOI:** 10.1007/s11524-026-01070-6

**Published:** 2026-04-13

**Authors:** Russell A. Brewer, Ellen Almirol, Makenna Meyer, Lin Tong, Sarah Hodge, Chandler Carter, Sierra Arnold, Lysa Vasquez, Alicia Dawdani, Jeannette Webb, Darjai Payne, Michelle M. Johns, Maria M. Pyra, Alida M. Bouris, En-Ling Wu, Gjvar Payne, Heather Horton, O’Dell Johnson, Amelia Knopf, Tamika C. Zapolski, Matthew C. Aalsma, Nickolas Zaller, John A. Schneider

**Affiliations:** 1 Department of Medicine, University of Chicago, Chicago, IL USA; 2 North Carolina State University, Raleigh, NC USA; 3 Biostatistics Lab, University of Chicago, Chicago, IL USA; 4 NORC at the University of Chicago, Chicago, IL USA; 5 Department of Medical Social Sciences, Northwestern University Feinberg School of Medicine, Chicago, IL USA; 6 Crown Family School of Social Work, Policy, and Practice, University of Chicago, Chicago, IL USA; 7 Capitol Area Reentry Program, Baton Rouge, LA USA; 8 University of Arkansas for Medical Sciences, Little Rock, AR USA; 9 School of Nursing, Indiana University, Indianapolis, IN USA; 10 School of Medicine, Indiana University, Indianapolis, IN USA; 11 The Health Alliance for Violence Intervention (HAVI), Boston, MA USA

**Keywords:** COVID-19, Interaction with law enforcement, Prevention, Social network, Group-level intervention, Criminal legal system

## Abstract

Social network diffusion interventions are a promising strategy for promoting health behavior change at the community level. Individuals impacted by the criminal legal system (CLS) experienced disproportionately high morbidity and mortality during the COVID-19 pandemic. As part of the National Institutes of Health Rapid Acceleration of Diagnostics–Underserved Populations (RADX-UP) initiative, we evaluated a group-level social network diffusion intervention incorporating motivational interviewing strategies to increase COVID-19 testing and vaccination among CLS-impacted individuals and their social networks across four urban cities. Between January 2023 and February 2024, participants were randomized by cohort to either a Motivational Interviewing–based intervention (MI) or a Prevention Education (PE) comparison condition. The MI intervention incorporated motivational interviewing–informed strategies to support participants in engaging and influencing members of their social networks, whereas PE participants received COVID-19 prevention education. The primary outcome was uptake of COVID-19 testing or vaccination within 90 days among previously unvaccinated primary participants, with testing and vaccination outcomes for social network members assessed based on participant report. Secondary outcomes included COVID-19 testing and vaccination knowledge among primary participants and reported resource sharing within social networks. A total of 810 primary participants were enrolled (MI: *n* = 403; PE: *n* = 407), with most identifying as Black or African American (89.8%) and 9.5% as Hispanic. At baseline, 83% of primary participants had previously been tested for COVID-19, and 70% had received at least one vaccine dose. At the 90-day follow-up, no statistically significant differences were observed between MI and PE groups in COVID-19 testing or vaccination uptake among primary participants or their network members. However, primary participants in the MI condition demonstrated significantly higher mean COVID-19 testing and vaccination knowledge scores compared with those in the PE condition. Patterns of resource sharing differed across study arms. Relative to MI, PE participants were more likely to share COVID-19 testing and vaccination resources at 30 days (OR = 0.54, 95% CI: 0.39–0.74, *p* < 0.01), though this difference was not sustained at 90 days (OR = 0.89, 95% CI: 0.58–1.35, *p* = 0.576). While the intervention did not produce significant differences in COVID-19 testing or vaccination uptake, both approaches facilitated dissemination of COVID-19–related resources and improved knowledge within CLS-impacted communities. These findings highlight both the potential and limitations of social network–based approaches for translating increased awareness into sustained preventive health behaviors.

**Supplementary Information:**

The online version contains supplementary material available at 10.1007/s11524-026-01070-6.

## Introduction

Criminal legal system (CLS)-impacted populations including individuals who are currently detained (e.g., in jails and prisons) as well as non-incarcerated individuals with a recent arrest, probation, parole, and/or involvement in diversion programs were disproportionately impacted by the COVID-19 pandemic [1–3]. Elevated risk in these populations has been attributed to structural factors such as overcrowded congregate settings, limited access to preventive healthcare, high prevalence of underlying health conditions, and disruptions in care continuity [1–4]. Despite expanded availability of COVID-19 testing and vaccinations, substantial hesitancy toward prevention services persisted, reflecting longstanding medical mistrust, experiences of stigma and discrimination, and systemic barriers to healthcare access [1–4].

Traditional public health messaging has often failed to address the specific concerns and barriers faced by CLS-impacted populations, which may further contribute to ongoing hesitancy toward COVID-19 testing and vaccination [3, 5, 6]. Health preventive decisions are shaped not only by individual knowledge but also by personal experiences, local disease prevalence, healthcare access, and structural factors [7–10]. As COVID-19 prevention guidance evolved towards more nuanced, risk-based recommendations such as the Centers for Disease Control and Prevention (CDC) emphasizing testing following symptoms or known exposure to COVID-19 [11], individuals increasingly relied on trusted sources within their social networks, including family members and close peers, for support and validation in health decision-making. These trusted social ties play a critical role in shaping health behaviors, particularly in addressing vaccine hesitancy and encouraging uptake of preventive measures [12].

The “Social Network Diffusion of COVID-19 Prevention for Diverse Criminal Legal Involved Communities” – the C3 Phase 2 study—leveraged this network-based approach through motivational interviewing strategies designed to mobilize participants’ organic social networks [13–15]. Social network diffusion interventions provide opportunities for behavior change through personally relevant and accessible channels within communities [16]. Beyond promoting testing and vaccination, the approach aims to foster community responsibility by empowering individuals to serve as health ambassadors within their networks, potentially amplifying prevention behaviors among both messengers and their contacts. This study builds on a growing body of research examining the effectiveness of social network diffusion interventions in reaching marginalized populations and addressing health disparities in complex, real-world settings [13, 14, 16].

## Methods

### Study Design

The C3 Phase 2 randomized clinical trial was conducted from January 2023 to May 2024 across four urban sites in the United States, a period marked by reduced population-level COVID-19 transmission and a corresponding decline in public urgency around testing and vaccination. All study procedures were approved by the appropriate Institutional Review Boards. The C3 Phase 2 study built on infrastructure developed from a Phase 1 study to address COVID-19 disparities among CLS-impacted populations [17]. C3 Phase 2 was a Type I network intervention focused on mobilizing participants' social networks through structured training [18]. A Type I network intervention refers to an approach that identifies and trains individuals within a social network to act as change agents—using their existing relationships to influence others and spread health or behavior change messages [18].

As part of Phase 2, primary participants were randomized to either an intervention arm, the Motivational Intervention (MI), or a control arm, Prevention Education (PE). The C3 Phase 2 MI curriculum consisted of four sequential learning modules delivered over a four-hour, group-based session. Sessions were facilitated by community members and trained interventionists with expertise in motivational interviewing techniques. The four modules included the following: (1) COVID-19 basics, including factual information and common myths; (2) COVID-19 testing and vaccination recommendations, with an emphasis on locally available resources; (3) real-life decision-making examples related to COVID-19 testing and vaccination, presented through personalized testimonial videos; and (4) social network–based motivation and skills building, including the development of “change agent” narratives and structured communication strategies for discussing COVID-19 testing and vaccination with friends and family members.

The MI sessions incorporated interactive activities and the same testimonial-based testing and vaccination videos used in the PE condition, ensuring consistency of informational content across study arms. The MI curriculum emphasized MI-informed strategies, such as values clarification, self-affirmation, and guided motivation for behavior change, to address misconceptions and barriers common among CLS-impacted populations and to promote adaptive COVID-19 prevention behaviors. Although participants were encouraged to engage members of their social networks around COVID-19 testing and vaccination, the intervention was not designed to train participants to independently deliver motivational interviewing techniques or to establish MI competency.

The PE condition served as an active control and was delivered in a group format by community members and trained interventionists. As with the MI arm, PE sessions consisted of four sequential learning modules delivered over a four-hour period. Modules included the following: (1) COVID-19 basics, including factual information and common myths; (2) COVID-19 testing and vaccination recommendations, with local resource information; (3) real-life decision-making examples related to COVID-19 testing and vaccination, presented through the same testimonial-based videos used in the MI condition; and (4) media literacy, focusing on skills to critically evaluate and interpret COVID-19 testing and vaccination information presented in the media.

Participants in the PE group were also encouraged to talk with friends, family members, and other social network contacts about COVID-19 testing and vaccination. However, unlike the MI condition, this encouragement occurred through general educational messaging rather than MI-informed facilitation or structured skill-building activities. Use of identical videos and shared encouragement to engage social networks across conditions allowed the study to isolate the added impact of MI-informed delivery strategies beyond shared content and expectations.

An active control condition was selected in lieu of clinic standard of care (i.e., provision of limited COVID-19 information and/or materials) for several reasons. First, standard of care would not adequately control for participant attention or intervention dosage, as both the MI and PE conditions required approximately four hours to deliver. Second, there is an ethical imperative to provide a robust and tailored control condition in the context of ongoing COVID-19 morbidity and mortality. Finally, community advisory board (CAB) guidance emphasized that participants should receive meaningful education and engagement in COVID-19 prevention as part of the research, rather than what could be perceived as substandard care.

To reinforce intervention content, participants in both study arms received a one-time booster session lasting approximately 20–30 min, delivered via phone or video call 30 days after completion of the initial intervention. Booster sessions in both groups included an assessment of participants’ engagement with social network members regarding COVID-19 testing and vaccination. The MI booster emphasized goal refinement and future-oriented planning, focusing on strengthening participants’ readiness and confidence to initiate or continue conversations with network members about COVID-19 testing and vaccination. This session included guided goal setting and preparation strategies consistent with MI-informed principles. In contrast, the PE booster was educational and reflective in nature, focusing on participants’ experiences discussing COVID-19 testing and vaccination with friends and family members. The PE booster emphasized reviewing what those conversations were like, addressing remaining questions, and providing clarification or additional information as needed, without structured goal-setting or motivational skill development.

#### Eligibility Criteria and Recruitment

Study participation at three of the four sites was open to individuals aged 18 or older who spent most of their time in Baton Rouge, LA; Chicago, IL; and Little Rock, AR. Additionally, a fourth site in Indianapolis, IN aimed to only recruit individuals 14 to 22 years of age. Minors were included in the study at the Indianapolis site to enhance inclusivity and provide insights to tailored public health strategies that address the specific needs and leverage the influential roles of youth and young adults in communities with significant law enforcement exposure that shape their health behaviors and trust in public health interventions [19]. However, due to implementation challenges, the Indianapolis site was unable to recruit sufficient youth and young adults for the group sessions (*n* = 2) and was therefore not included in the analyses.

The study focused on community-dwelling CLS-impacted individuals rather than individuals who were currently incarcerated. This decision was informed by ethical, logistical, and scientific considerations, as well as pandemic-related access constraints. During the COVID-19 pandemic, research access to jails and prisons was severely restricted, limiting the feasibility of enrolling incarcerated individuals and delivering group-based interventions within custodial settings.

In addition to these access limitations, individuals who are incarcerated are afforded additional human subjects protections as a vulnerable population, and carceral environments impose substantial restrictions on intervention delivery, communication, and follow-up. Moreover, the intervention was designed to leverage community social networks to diffuse COVID-19 prevention information and resources—an approach that is inherently constrained in correctional settings, where social ties are limited and testing and vaccination decisions are often institutionally driven. Focusing on non-incarcerated CLS-impacted individuals therefore allowed for greater feasibility, flexibility, and ethical rigor.

Additional eligibility criteria required participants to complete the study in English and to report either direct or witnessed exposure to law enforcement, including being personally stopped, searched, or arrested and/or witnessing someone else being stopped, searched, or arrested. The inclusion of individuals with indirect exposure was critical, as witnessing law enforcement interactions, particularly those involving family members, peers, or community members, can have significant psychological, social, and behavioral impacts [20]. Such experiences contribute to broader perceptions of surveillance, mistrust, and systemic harm, which are important factors influencing health behaviors and access to services. To enhance the reach and potential impact of the study, individuals with prior COVID-19 vaccination or infection history were eligible for participation, recognizing their potential role as network change agents capable of sharing accurate information and resources within their social networks.

Exclusion criteria included an inability to provide informed consent and conduct the study in English, lack of access to a phone, not being able to commit 90 days to participate in the study, reported presence of active COVID-19 symptoms (as defined by CDC guidelines) [11], currently detained in jail or prison, currently residing in a court-ordered treatment center as part of parole, and current placement in foster care (Indiana site only).

Potential participants were recruited by local research assistants embedded within community settings that provided in-person and remote social and care services, including community COVID-19 testing and vaccination sites. Recruitment strategies included in-person recruitment at study centers, flyer distribution in community spaces, social media postings, and contacting individuals from previous studies who had indicated interest in future research. Participants received up to $200 for participating in the study.

### Randomization

Randomization occurred as a stratified block design to ensure balanced allocation across sites. Utilizing Research Electronic Data Capture (REDCap) randomization module, participants were assigned in cohorts of 8–23 individuals to the intervention, i.e., MI or PE, where a cohort would be assigned a single study assignment.

### Data Collection

Quantitative data were collected through REDCap, a secure web-based data collection platform. Trained research assistants administered three surveys via tablet devices during in-person sessions and through telephone/video calls at baseline and follow-ups at 30–90 days post-intervention. Network member outcomes such as COVID-19 testing and vaccination history were also reported by primary participants. REDCap’s mobile app facilitated offline data collection, when necessary, with automatic synchronization once internet connectivity was restored.

#### Study Measures

Baseline assessments included demographics, highest level of education completed, employment status, CLS interactions, and COVID testing and vaccination history [21]. At baseline, participants were asked to share up to 10 contacts of who they would discuss COVID testing and vaccination resources with after the intervention and were allowed to include an additional 10 contacts at the 30-day follow-up.

#### Study Outcomes

Primary outcomes were defined as (1) receipt of at least one COVID-19 test within 90 days of baseline and (2) receipt of at least one COVID-19 vaccine dose within 90 days of baseline among previously unvaccinated primary participants. These outcomes were ascertained through participant self-report and, when available, verified using city and state registry data. Participants were also able to provide documentation, such as test results or vaccination records, during follow-up surveys conducted at 30 and 90 days. COVID-19 testing and vaccination behaviors among network members were collected solely through reports provided by primary participants and were not independently verified.

Secondary outcomes included COVID-19 testing and vaccination knowledge among primary participants, assessed using a predetermined 16-item questionnaire with response options of *true*, *false*, or *don’t know* [21]. Items assessed common misconceptions and factual understanding of COVID-19 prevention. Examples statements included, *“I don’t need to get a COVID-19 test unless I feel sick”* or *“If I already had COVID-19, I do not need to get the vaccine”*. Participants completed the questionnaire at baseline and at 30- and 90-day follow-up assessments. Each item had a single correct response based on current public health guidance. Responses were scored dichotomously: correct responses were coded as 1, and incorrect or “don’t know” responses were coded as 0. An overall mean knowledge score was calculated for each participant by averaging scores across all 16 items, with higher values indicating greater COVID-19 testing and vaccination knowledge. Mean knowledge scores and standard deviations (SD) were calculated for each study group at baseline, 30 days, and 90 days. Changes in mean knowledge scores over time and differences between intervention and comparison groups were used to evaluate the effect of the intervention on COVID-19–related knowledge. An additional secondary outcome was resource sharing, defined as whether primary participants reported sharing information about community-based COVID-19 testing and vaccination resources with members of their social networks at the 30- and 90-day follow-up assessments, with a binary response “yes” or “no” for each mentioned network member [21].

### Statistical Analyses

Baseline characteristics were summarized for the overall sample and stratified by intervention and control groups using *t* tests for continuous variables (e.g., age) and χ^2^ tests or Fisher’s exact tests for categorical variables, as appropriate. The primary analysis compared receipt of at least one COVID-19 test and/or COVID-19 vaccination over the 90-day follow-up period between intervention and control groups. Outcomes were assessed separately among primary participants and network members and compared by intervention group at each time point (baseline, 30 days, and 90 days). Analyses included data from Baton Rouge, LA; Chicago, IL; and Little Rock, AR; the Indianapolis site was excluded.

For the COVID-19 testing outcome, the full sample was included in the analyses (*n* = 810 primary participants; *n* = 1626 network participants), as participants could obtain a COVID-19 test during follow-up regardless of prior testing history. For the vaccination outcome, analyses were restricted to participants who had not received any COVID-19 vaccine dose at or before baseline, because the outcome reflected new vaccine uptake during the follow-up period. This resulted in a final analytic sample of 565 primary participants and 911 network participants.

Longitudinal mixed-effects models were used to assess the effect of the intervention on COVID-19 testing and vaccination outcomes over time. Mixed-effects logistic regression models were fitted for the binary outcomes and included fixed effects for intervention group, time, and the intervention-by-time interaction. Time was modeled as a categorical variable corresponding to assessment points at baseline, 30 days, and 90 days (Fig. 2). All models accounted for within-participant correlation due to repeated measures by including random intercepts; random slopes for time were evaluated and included when supported by model fit.

Study site was included as a fixed effect based on post hoc sensitivity analyses examining baseline differences in outcomes by site among primary participants and network members (Supplementary Tables 1 and 2). These sensitivity analyses were descriptive in nature and were conducted using the full available sample; outcome-specific exclusion criteria (e.g., prior COVID-19 vaccination) were not applied, as the purpose was to assess site-level differences rather than intervention effects. All analyses were conducted using Stata/SE version 19 and R for data management, descriptive analyses, and figure generation.

## Results

**Fig. 1:**
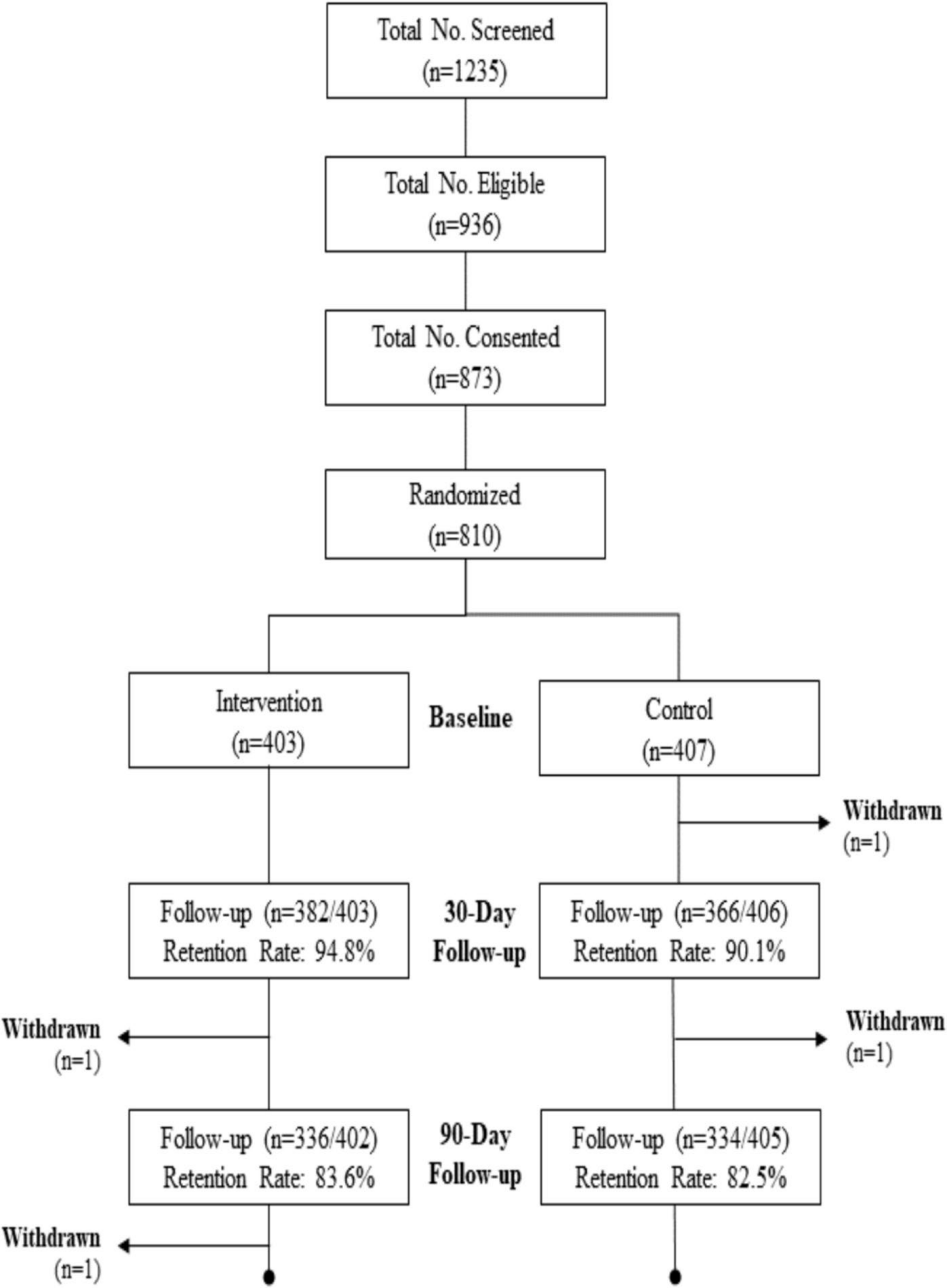
Consort diagram of the C3 phase 2 study conducted from 2023 to 2024

A total of 810 participants were enrolled and randomized to either the MI group (*n* = 403) or the PE control group (*n* = 407) across three study sites (Fig. 1). Baseline primary participant characteristics by condition are described in Table 1. The mean age was 42.9 years (SD = 14.6). Most participants identified as Black or African American (89.8%), with 9.5% as Hispanic. Educational attainment was high school or some college for 71% of participants. Nearly half identified as cisgender women (49%) and 46% as cisgender men. Approximately 30% of participants were employed, and the majority identified as heterosexual (68%). Nearly three-quarters of participants (72%) reported prior direct interactions with law enforcement, and 96% reported having witnessed interactions with law enforcement. Approximately one-third of participants were recruited from Chicago and Baton Rouge, respectively, with an additional 25% recruited from Little Rock. At baseline, 83% had previously been tested for COVID-19, and 70% had received at least one COVID vaccine dose. There were no statistically significant differences in baseline characteristics between study conditions, with the exception of race; a higher proportion of participants identifying as Black were assigned to the intervention condition compared with the control condition (Table 1).

**Table 1:** Baseline C3 phase 2 participant characteristics by condition (*N* = 810)

	Total *N* = 810	Intervention *n* = 403	Control *n* = 407	*P*-value
*Demographics*				
Age, mean (SD)	42.9 (14.6)	42.9 (14.6)	43.0 (14.6)	0.903
Race, Black or African American	727 (89.8%)	374 (92.8%)	353 (86.7%)	** < 0.01**
Ethnicity, non-Hispanic	733 (90.5%)	371 (92.1%)	362 (89.2%)	0.313
Gender identity				0.306
Cisgender man	375 (46.3%)	197 (48.9%)	178 (43.7%)	
Cisgender woman	400 (49.4%)	191 (47.4%)	209 (51.4%)	
Transgender man	7 (0.9%)	1 (0.2%)	6 (1.5%)	
Transgender women	9 (1.1%)	5 (1.2%)	4 (1.0%)	
Non-binary/other	11 (1.4%)	6 (1.5%)	5 (1.2%)	
PNTA/missing	8 (1.0%)	3 (0.7%)	5 (1.2%)	
Sexual orientation				0.863
Gay/lesbian	124 (15.3%)	63 (15.6%)	61 (15.0%)	
Bisexual	76 (9.4%)	42 (10.4%)	34 (8.4%)	
Heterosexual	552 (68.2%)	271 (67.2%)	281 (69.2%)	
Other	18 (2.2%)	8 (2.0%)	10 (2.5%)	
PNTA/missing	39 (4.8%)	19 (4.7%)	20 (4.9%)	
Educational attainment				0.927
Less than high school	147 (18.2%)	73 (18.1%)	74 (18.2%)	
HS graduate/some college	572 (70.7%)	283 (70.2%)	289 (71.2%)	
Bachelor’s or higher	71 (8.8%)	38 (9.4%)	33 (8.1%)	
Missing	19 (2.3%)	9 (2.2%)	10 (2.5%)	
Employed	233 (28.8%)	123 (30.5%)	110 (27.0%)	0.307
*Criminal legal system interaction*				
Interacted with law enforcement	579 (71.5%)	291 (72.2%)	288 (70.8%)	0.705
Witnessed interaction with law enforcement	779 (95.7%)	386 (95.8%)	393 (96.6%)	0.693
*COVID-19 behaviors*				
Tested for COVID-19, prior to baseline	670 (82.8%)	331 (82.1%)	339 (83.5%)	0.873
Vaccinated against COVID-19	565 (69.8%)	272 (67.5%)	293 (72.0%)	0.507
Study sites				0.928
Chicago, IL	309 (38.2%)	154 (38.2%)	155 (38.1%)	
Little Rock, AR	205 (25.3%)	104 (25.8%)	101 (24.8%)	
Baton Rouge, LA	296 (36.5%)	145 (36.0%)	151 (37.0%)	

Bold indicates a *p*-value < 0.05. Missing data greater than 10%: age (*n* = 160, 20.5%); PNTA=Prefer not to answer

### Primary Outcomes

**Fig. 2:**
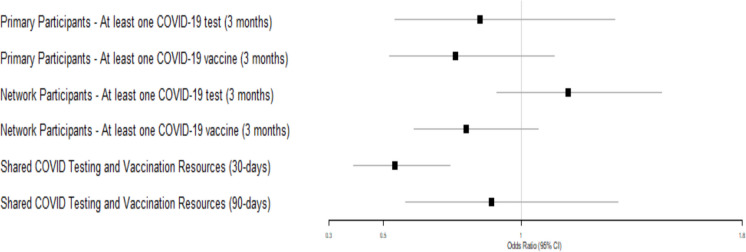
Primary and secondary outcomes: intervention effects on COVID-19 testing, vaccination, and shared resources. Note: forest plot showing odds ratios (ORs) and 95% confidence intervals (CIs). Vertical dashed line represents null effect (OR = 1.0). Primary outcomes: COVID testing and vaccination uptake within 3 months of baseline comparing intervention (MI) and control (PE) groups among primary participants. Secondary outcomes: COVID testing and vaccination uptake within 3 months of baseline comparing intervention (MI) and control (PE) groups among network participants; shared resources are defined by whether the primary participant shared COVID testing and vaccination resources with their network members at 30 and 90 days

Table 2 presents unadjusted comparisons between the intervention and control conditions at each assessment time point, reported separately for primary participants and their network members. No statistically significant differences between study conditions were observed at any time point for either group. Consistent with the unadjusted comparisons, longitudinal adjusted analyses (Fig. 2) demonstrated no statistically significant differences between intervention and control conditions for COVID-19 testing (OR = 0.85, 95% CI: 0.54–1.34; *p* = 0.49) or vaccination (OR = 0.76, 95% CI: 0.52–1.12; *p* = 0.17) among primary participants. Similarly, no significant differences by study condition were observed among network members for COVID-19 testing (OR = 1.17, 95% CI: 0.98–1.51; *p* = 0.22) or vaccination (OR = 0.80, 95% CI: 0.61–1.06; *p* = 0.12).

**Table 2:** Unadjusted COVID-19 testing and vaccination outcomes of primary participants and network members at 30 and 90 days, by study condition

	Total		Intervention	Control	*p*-value
Primary participants	*N* = 810		*N* = 403	*N* = 407	
COVID tested^1^					
Baseline	670 (85.4%)		331 (84.4%)	339 (86.3%)	0.471
30 days	103 (14.2%)		50 (14.0%)	53 (14.4%)	0.890
90 days	170 (26.2%)		90 (27.4%)	80 (25.0%)	0.480
COVID vaccinated^2^					
Baseline	565 (69.8%)		272 (67.5%)	293 (72.0%)	0.164
30 days	37 (5.1%)		19 (5.4%)	18 (4.9%)	0.779
90 days	47 (7.3%)		24 (7.4%)	23 (7.3%)	0.923
Network members		*N* = 1626**	*N* = 765	*N* = 861	
COVID tested^3^					
Baseline	722 (45.1%)		357 (47.5%)	365 (43.0%)	0.075
30 days	277 (29.8%)		120 (28.4%)	157 (31.0%)	0.401
90 days	174 (34.9%)		85 (33.5%)	89 (36.3%)	0.502
COVID vaccinated^4^					
Baseline	911 (56.7%)		430 (56.8%)	481 (56.6%)	0.931
30 days	175 (18.9%)		78 (18.5%)	97 (19.3%)	0.768
90 days	118 (23.5%)		56 (21.8%)	62 (25.4%)	0.340

Total *N* (i.e., denominators)

^1^Baseline, *n* = 785; 30 days, *n* = 726, 90 days, *n* = 648

^2^Baseline, *n* = 810; 30 days, *n* = 723, 90 days, *n* = 647

^3^Baseline, *n* = 1600; 30 days, *n* = 929, 90 days, *n* = 499

^4^ Baseline, *n* = 1607; 30 days, *n* = 926, 90 days, *n* = 501

^**^Individuals who were reported by a primary participant, includes up to 20 network members (*N* = 1626)

Unadjusted site-level analyses presented in Supplementary Table 1 showed consistently higher COVID-19 testing rates at Site 1 compared with the other two sites among both primary participants and network members across all three assessment time points. While Supplementary Table 2 similarly indicated higher COVID-19 vaccination uptake at Site 1 relative to the other sites among network members at baseline and 30 days.

### Secondary Outcomes

With respect to COVID-19 testing and vaccination knowledge, participants in the MI group had a significantly higher mean knowledge score compared with those in the PE group (13.1 vs. 12.6, *p* = 0.009) (Table 3). This finding suggests that the MI intervention was associated with greater understanding of COVID-19 testing and vaccination concepts relative to the PE condition, although the magnitude of the difference was small.

**Table 3:** Primary participant COVID-19 knowledge by intervention (MI) and control (PE) conditions, at baseline, 30 days, and 90 days

	Baseline		30 days		90 days	
	Intervention	Control	Intervention	Control	Intervention	Control
I can get COVID-19 by getting tested for it	87.2%	85.4%	92.7%	89.7%	93.9%	91.2%
I should get tested if I was in close contact with someone who was infected with COVID-19	84.1%	82.5%	94.4%	93.2%	94.5%	95.0%
An antibody test can tell me whether I am currently infected with COVID-19	53.4%	57.9%	37.8%	41.2%	43.3%	37.5%
I do not need to get a COVID-19 test unless I feel sick.^1^	80.3%	79.7%	83.8%	80.0%	**86.3%**	**80.6%**
Young people infected with COVID-19 can get seriously ill and even die from it	64.7%	64.2%	88.1%	84.3%	87.8%	87.2%
The COVID-19 vaccine ingredients are safe to put into my body	37.4%	38.3%	63.0%	62.2%	61.7%	63.4%
The COVID-19 vaccines came out so fast because they were not carefully tested.^2^	**73.0%**	**79.8%**	71.9%	73.3%	72.8%	72.0%
I can get COVID-19 by getting vaccinated for it	83.1%	83.6%	89.3%	87.3%	90.4%	85.6%
If I already had COVID-19, I do not need to get the vaccine	87.8%	90.2%	93.8%	90.0%	91.7%	89.7%
If I eat right, exercise, and take good care of my body, I do not need to worry about getting COVID-19.^3^	87.3%	88.1%	91.3%	91.1%	**95.1%**	**89.7%**
COVID-19 vaccines can alter my DNA	90.4%	90.2%	91.6%	91.9%	90.5%	86.6%
COVID-19 vaccines will affect my fertility	90.9%	91.4%	90.4%	89.4%	89.5%	90.0%
Children as young as 2 can get a COVID-19 vaccine	32.3%	31.1%	64.0%	59.4%	65.6%	62.2%
Immunocompromised individuals are more likely to get very sick from COVID-19.^4^	42.8%	41.0%	74.2%	71.7%	**77.1%**	**69.8%**
COVID-19 can be transmitted through close contact with an infected person who has symptoms	66.3%	65.2%	88.8%	86.0%	87.2%	83.3%
COVID-19 can be transmitted through close contact with an infected person even if they are not showing symptoms of infection	65.5%	66.0%	88.8%	85.4%	88.7%	84.0%
COVID-19 knowledge score, mean, (SD)	11.2 (2.3)	11.2 (2.6)	13.0 (2.2)	12.7 (2.2)	**13.1 (2.1)**	**12.6 (2.4)**

Bold indicates a *p*-value less than 0.05. *p*-value = 0.009 at 90 days between MI (13.1, SD 2.1) vs. PE (12.6, SD 2.4)

^1^*p*-value = 0.050 at 90 days between MI (86.3%) and PE (80.6%)

^2^*p*-value = 0.025 at baseline between MI (73.0%) and PE (79.8%)

^3^*p*-value = 0.009 at 90 days between MI (95.1%) and PE (89.7%)

^4^*p*-value = 0.036 at 90 days between MI (77.1%) and PE (69.8%)

In terms of individual knowledge items, a significant between-group difference in the proportion of correct responses was observed for the item stating that *“COVID-19 vaccines came out so fast because they were not carefully tested,”* with a lower percentage of correct responses in the PE condition compared with the MI condition (PE: 79.8% vs. MI: 73%; *p* = 0.025). This difference was no longer evident at the 30- or 90-day follow-up assessments, with endorsement of this belief converging to approximately 73% across both conditions. No other knowledge differences between study conditions were observed at baseline or at 30 days.

At the 90-day follow-up, three statistically significant item-level differences were observed between study conditions. A greater proportion of participants in the MI condition correctly responded that *“I don’t need to get a COVID-19 test unless I feel sick”* is false (MI: 86.3% vs. PE: 80.6%; *p* = 0.050) and that *“immunocompromised individuals are more likely to get very sick from COVID-19”* is true (MI: 77.1% vs. PE: 69.8%; *p* = 0.036). In contrast, a higher proportion of MI participants incorrectly endorsed the misconception that *“If I eat right, exercise, and take good care of my body, I don’t need to worry about getting COVID-19”* compared with participants in the PE condition (MI: 95.1% vs. PE: 89.7%; *p* = 0.009). No additional statistically significant differences in knowledge were observed between study conditions at the 90-day follow-up.

In analyses of resource sharing, a significant difference was observed at the 30-day follow-up: participants in the MI condition had lower odds of sharing COVID-19 testing and vaccination resources with network members compared with those in the PE condition (OR = 0.54, 95% CI: 0.39–0.74, *p* < 0.01). This difference was no longer observed at the 90-day follow-up (OR = 0.89, 95% CI: 0.58–1.35, *p* = 0.576) (data not shown in Tables).

## Discussion

Although no differences were observed between the MI and PE groups in COVID-19 testing or vaccination uptake among primary participants or their network members, participants in the PE arm had higher odds of sharing COVID-19 testing resources at the 30-day follow-up relative to those in the MI arm; however, this difference was not sustained at 90 days. One possible explanation for this short-term pattern is that the PE curriculum placed greater emphasis on concrete information dissemination—such as sharing local testing locations and resources—which may have facilitated immediate, low-burden sharing behaviors within participants’ social networks. In contrast, the MI intervention emphasized internal motivation, values clarification, and skill-building for conversations, which may be more conducive to complex or sustained behavior change but less likely to produce immediate resource-sharing behaviors.

Importantly, the study was conducted during a period of lower community transmission and reduced public urgency surrounding COVID-19 testing and vaccination, which may have further favored brief, information-based sharing over sustained engagement. Nonetheless, the intervention was intentionally designed to address diminished risk perception, misinformation, and pandemic fatigue through motivational interviewing–informed approaches and social network–based strategies. Evaluating intervention effects under these conditions enhances the relevance of the findings to the current endemic phase of COVID-19 prevention, in which ongoing engagement rather than crisis-driven response is increasingly required.

Overall, the intervention was associated with modest but statistically significant improvements in COVID-19 testing and vaccination knowledge, with participants in the MI condition demonstrating higher mean knowledge scores compared with those in the PE condition. These findings suggest that MI may be more effective than passive educational approaches in improving factual understanding of COVID-19–related prevention and vaccination information.

At the item level, MI participants were more likely at 90 days to correctly reject the belief that COVID-19 testing is unnecessary in the absence of symptoms and to recognize that immunocompromised individuals are at increased risk for severe COVID-19 illness. These findings are particularly notable given the continued importance of asymptomatic testing and protection of high-risk populations and suggest that MI may support sustained understanding of key public health messages. However, differences were also observed in the endorsement of specific beliefs across study conditions. In particular, a higher proportion of MI participants endorsed the belief that healthy lifestyle behaviors alone are sufficient to prevent COVID-19 infection. This pattern underscores the challenge of addressing beliefs related to personal health and immunity and suggests that some aspects of COVID-19 risk perception may be resistant to change, even in interventions that incorporate motivational and skills-based components.

Importantly, the magnitude of between-group differences in knowledge was small, suggesting that while MI can improve knowledge, knowledge gains alone may be insufficient to meaningfully shift health-related beliefs or translate into behavior change [22]. These findings align with prior research indicating that multifaceted interventions addressing social norms, structural barriers, and exposure to misinformation may be necessary to achieve sustained improvements in COVID-19–related behaviors [8–10].

Despite the absence of significant differences in the primary outcomes, both MI and PE demonstrated value by promoting resource sharing among primary participants in both groups, indicating that participants felt empowered to serve as credible sources of information within their social networks. These findings extend prior work by demonstrating that network-based interventions can mobilize information and resource sharing within CLS-impacted populations, even when immediate behavior change is not observed. Such interventions may therefore serve as an important mechanism for building community resilience and expanding access to health-related resources over time.

Overall employment levels among participants were low, reflecting the persistent structural barriers to stable employment experienced by CLS-impacted populations. Low employment may have influenced participants’ ability to engage in COVID-19 testing and vaccination by limiting access to employer-facilitated testing, paid time off, and employer-sponsored health insurance, as well as by competing with more immediate priorities related to housing and economic insecurity. These constraints may help explain why improvements in COVID-19 knowledge and resource sharing did not translate into measurable increases in testing or vaccination uptake, highlighting the limits of information- and motivation-focused interventions in the absence of broader structural supports.

Several limitations warrant consideration. Most notably, the absence of a standard control group limits causal inference regarding intervention effectiveness. While inclusion of a usual-care or non-intervention control condition would typically strengthen internal validity, such an approach was considered ethically inappropriate during a public health emergency.

High baseline levels of COVID-19 testing (83%) and vaccination (70%) among participants may have introduced ceiling effects, limiting the ability to detect additional gains and potentially attenuating observed intervention effects. Consequently, the observed effects may underestimate the true impact of the intervention, particularly for outcomes with limited variability.

An additional limitation is that network-level outcomes, including resource sharing as well as network members’ COVID-19 testing and vaccination behaviors, were based on self-reported information provided by primary participants and were not independently verified. Primary participants may not have had complete or accurate knowledge of network members’ behaviors, and responses may have been influenced by recall or social desirability bias. As a result, estimates of intervention effects on network-level outcomes should be interpreted with caution. Although reliance on participant report is common in social network–based intervention research and was the most feasible approach given study design and ethical considerations, future studies could strengthen measurement validity by incorporating direct assessment of network members, linkage to administrative or registry data where feasible, or alternative approaches to capturing peer influence and information diffusion.

Measurement reactivity related to the assessment of social network engagement at the 30-day booster session is also possible. However, encouragement to engage network members was embedded in both study conditions, and assessments occurred at identical time points across arms. To the extent that measurement reactivity occurred, it would likely be non-differential and bias results toward the null, suggesting that observed between-group differences may represent conservative estimates of intervention effects.

Although eligibility criteria were intentionally broad to capture a range of CLS experiences, the study assessed only two forms of CLS exposure at baseline: direct interaction with law enforcement and witnessing an interaction with law enforcement. More granular information regarding other types of CLS involvement—such as incarceration, probation or parole, court involvement, or the duration and recency of legal system contact—was not collected. In addition, there was substantial overlap between participants reporting direct and witnessed law enforcement interactions, which limited variability in CLS exposure categories. As a result, we were unable to examine heterogeneity of intervention effects by type, intensity, or duration of CLS exposure. Future studies that more comprehensively characterize CLS involvement may be better positioned to assess whether intervention effectiveness differs across specific forms of legal system contact and to identify subpopulations for whom tailored approaches may be most impactful.

The use of phone- and video-based data collection may have introduced measurement error related to technology access or privacy constraints. Because data collection procedures were identical across study conditions, any such effects are likely to have been non-differential. Additionally, site-specific variability in engagement and outcomes underscores the influence of local context—including trust in public health initiatives and access to supportive services—on intervention effectiveness and limits generalizability.

Finally, although the original study design included verification of testing and vaccination outcomes through city and state registry data, administrative delays and incomplete reporting at one implementation site necessitated reliance on self-reported outcomes for primary analyses. One site was unable to provide any laboratory-confirmed testing or vaccination data for enrolled participants, which may have resulted in underestimation of testing or vaccination uptake. In addition, the study did not include formal assessments of participant learning or acquisition of MI-related skills, limiting the ability to directly examine mechanisms of behavior change.

## Conclusion

This study contributes to the growing literature on social network–based interventions by demonstrating that peer-driven approaches can facilitate COVID-19–related information and resource sharing within CLS-impacted communities, even in the absence of measurable changes in COVID-19 testing or vaccination uptake. These findings highlight the value of leveraging social networks to support community-level engagement while also underscoring the limitations of network-based strategies when structural barriers to healthcare access remain unaddressed. Although the intervention did not yield significant effects on primary outcomes, dissemination of these findings is critical to advancing scientific understanding, reducing publication bias, and informing the design of future public health interventions. Collectively, these results emphasize the importance of multi-component strategies that integrate social network approaches with structural supports to more effectively promote preventive health behaviors among CLS-impacted populations. A separate manuscript detailing the implementation evaluation is currently in development and will further elucidate contextual and process-related factors influencing intervention delivery and outcomes.

## Supplementary Information

Below is the link to the electronic supplementary material.

ESM 1 (DOCX 24.4 KB)

## Data Availability

The de-identified common data elements used in this study are available through the RADx Data Hub at https://radxdatahub.nih.gov/. Access to the data is subject to approval in accordance with NIH data access policies.
