# Supplementary material for: Effectiveness Outcomes from a Social Network Diffusion Intervention to Increase COVID-19 Testing and Vaccination among Individuals Impacted by the Criminal Legal System in Urban U.S. Cities
Source: J Urban Health. 2026 Apr 13;103(2):454–67. doi: 10.1007/s11524-026-01070-6 (PMC13126537; doi:10.1007/s11524-026-01070-6)
Supplement: Supplementary file 1 — (DOCX 24.4 KB) [file 11524_2026_1070_MOESM1_ESM.docx]

**Supplementary** **Table 1.** Primary Outcomes of COVID Testing, by Study Site at each time point

| **COVID-19 test** | **Total** | **COVID Test** | **No COVID Test** | ***p-value*** |
| --- | --- | --- | --- | --- |
| Primary Participants |  |  |  |  |
| Baseline |  |  |  |  |
| Site 1 | 303 (100%) | 274 (90.4%) | 29 (9.6%) | **0.006** |
| Site 2 | 199 (100%) | 164 (82.4%) | 35 (17.6%) |  |
| Site 3 | 283 (100%) | 232 (82.0%) | 51 (18.0%) |  |
| By 30 days |  |  |  |  |
| Site 1 | 270 (100%) | 59 (21.9%) | 211 (78.2%) | **0.000** |
| Site 2 | 194 (100%) | 13 (6.7%) | 181 (93.3%) |  |
| Site 3 | 262 (100%) | 31 (11.8%) | 231 (88.2%) |  |
| By 90 days |  |  |  |  |
| Site 1 | 237 (100%) | 83 (35.0%) | 154 (65.0%) | **0.000** |
| Site 2 | 178 (100%) | 44 (24.7%) | 134 (75.3%) |  |
| Site 3 | 233 (100%) | 43 (18.5%) | 190 (81.6%) |  |
| Network Members |  |  |  |  |
| Baseline |  |  |  |  |
| Site 1 | 683 (100%) | 340 (49.8%) | 343 (50.2%) | **0.000** |
| Site 2 | 471 (100%) | 172 (36.5%) | 299 (63.5%) |  |
| Site 3 | 446 (100%) | 210 (47.1%) | 236 (52.9%) |  |
| By 30 days |  |  |  |  |
| Site 1 | 425 (100%) | 146 (34.4%) | 279 (65.6%) | **0.000** |
| Site 2 | 280 (100%) | 57 (20.4%) | 223 (79.6%) |  |
| Site 3 | 224 (100%) | 74 (33.0%) | 150 (67.0%) |  |
| By 90 days |  |  |  |  |
| Site 1 | 226 (100%) | 94 (41.6%) | 132 (58.4%) | **0.016** |
| Site 2 | 152 (100%) | 44 (29.0%) | 108 (71.0%) |  |
| Site 3 | 121 (100%) | 36 (29.8%) | 85 (70.3%) |  |
| **Bold** indicates a p-value <0.05. Total = Includes number of primary participants and reported networked members tested at each time point. Outcome-specific analytic exclusions (e.g., prior vaccination) were not applied for this descriptive sensitivity analysis. | | | | |
|  | | | | |

**Supplementary** **Table 2.** Primary Outcomes of COVID Vaccination, by Study Site at each time point

| **At least one COVID-19 vaccine dose** | **Total** | **COVID Vaccination** | **No COVID Vaccination** | ***p-value*** |
| --- | --- | --- | --- | --- |
| Primary Participants |  |  |  |  |
| Baseline |  |  |  |  |
| Site 1 | 309 (100%) | 215 (69.6%) | 94 (30.4%) | 0.905 |
| Site 2 | 205 (100%) | 141 (68.8%) | 64 (31.2%) |  |
| Site 3 | 296 (100%) | 209 (70.6%) | 87 (29.4%) |  |
| By 30 days |  |  |  |  |
| Site 1 | 269 (100%) | 13 (4.8%) | 256 (95.2%) | 0.081 |
| Site 2 | 193 (100%) | 5 (2.6%) | 188 (97.4%) |  |
| Site 3 | 261 (100%) | 19 (7.3%) | 242 (92.7%) |  |
| By 90 days |  |  |  |  |
| Site 1 | 237 (100%) | 11 (4.6%) | 226 (95.4%) | 0.147 |
| Site 2 | 178 (100%) | 16 (9.0%) | 162 (91.0%) |  |
| Site 3 | 232 (100%) | 20 (8.6%) | 212 (91.4%) |  |
| Network Members |  |  |  |  |
| Baseline |  |  |  |  |
| Site 1 | 689 (100%) | 366 (53.1%) | 323 (46.9%) | **0.000** |
| Site 2 | 466 (100%) | 251 (53.9%) | 215 (46.1%) |  |
| Site 3 | 452 (100%) | 294 (65.0%) | 158 (35.0%) |  |
| By 30 days |  |  |  |  |
| Site 1 | 425 (100%) | 66 (15.5%) | 359 (84.5%) | **0.001** |
| Site 2 | 278 (100%) | 48 (17.3%) | 230 (82.7%) |  |
| Site 3 | 223 (100%) | 61 (27.4%) | 162 (73.6%) |  |
| By 90 days |  |  |  |  |
| Site 1 | 227 (100%) | 57 (25.1%) | 170 (74.9%) | 0.413 |
| Site 2 | 152 (100%) | 30 (19.7%) | 122 (80.3%) |  |
| Site 3 | 122 (100%) | 31 (25.4%) | 91 (74.6%) |  |
| **Bold** indicates a p-value <0.05. Total = Includes number of primary participants and reported networked members vaccinated at each time point. Outcome-specific analytic exclusions (e.g., prior vaccination) were not applied for this descriptive sensitivity analysis. | | | | |
